# Comparison of corneal and lens density measurements obtained by Pentacam and CASIA2 in myopes

**DOI:** 10.1186/s12886-023-03199-3

**Published:** 2023-11-10

**Authors:** Yijia Xu, Yuhao Ye, Yiyong Xian, Lingling Niu, Xingtao Zhou, Jing Zhao

**Affiliations:** 1 Department of Ophthalmology, NHC Key Laboratory of Myopia, Laboratory of Myopia, Eye and ENT Hospital of Fudan University, Chinese Academy of Medical Sciences, 83 Fenyang Road, Shanghai, 200031 China; 2 NHC Key Laboratory of Myopia (Fudan University), Key Laboratory of Myopia, Chinese Academy of Medical Sciences, Shanghai, China; 3 Shanghai Research Center of Ophthalmology and Optometry, Shanghai, China; 4 Shanghai Engineering Research Center of Laser and Autostereoscopic 3D for Vision Care, Shanghai, China

**Keywords:** Myopia, Corneal density, lens density, Pentacam, CASIA2

## Abstract

**Purpose:**

To investigate the agreement between Pentacam and CASIA2 in the evaluation of corneal densities (CDs) and lens densities (LDs) in myopes.

**Methods:**

Fifty-three patients (106 eyes) underwent comprehensive ophthalmologic examinations. CDs and LDs were measured using Pentacam and CASIA2, respectively, based on the grayscale percentage of the obtained images. Agreement between Pentacam and CASIA2 was evaluated using the consistency intraclass correlation coefficient (ICC) and represented using Bland-Altman plots.

**Results:**

Compared to Pentacam, CASIA2 showed significantly higher CD and LD values in all measured zones. The ICC of the average CD and LD measured by the Pentacam and CASIA2 were 0.726 and 0.757, respectively. The ICC values of all corneal zones and lenses were above 0.7, except for the measurement of the cornea in the 0–2 mm zone (0.455), suggesting good consistency between the two devices, whose results were of different levels of linear correlation. Bland-Altman plots showed mean percentages of 3.93% for the points falling outside the limits of agreement among the densitometry results. The ICCs in different age groups were similar, but the agreement was poorer in the high myopia group (low and moderate myopia, CD: 0.739, LD: 0.753; high myopia, CD: 0.621, LD: 0.760).

**Conclusions:**

CASIA2 demonstrated good consistency with Pentacam in the measurement of CD and LD, except for measurement of CD in the central cornea and in high myopia. Despite difference in the numerical results compared with Pentacam, which made the two devices uninterchangeable, CASIA2 provides a reliable alternative densitometric measurement method.

**Supplementary Information:**

The online version contains supplementary material available at 10.1186/s12886-023-03199-3.

## Introduction

The cornea and lens are important elements of the refractive components of the eye. When certain pathologic conditions occur in the cornea or lens, decreased transparency and increased light scattering can occur [1]. These changes in corneal health reflect changes in corneal density (CD). Therefore, CD can assist in the diagnosis of multiple keratopathy, the monitoring of corneal conditions after refractive surgery [2], and the prediction of visual acuity [3] as well as the visual quality [4]. The clarity and density of the lens are also crucial for maintaining high-quality vision. As cataract is the main cause of blindness in less developed countries [5], objective and quantifiable evaluation of lens density (LD) has become an essential clinical practice. As the number of patients undergoing implantable collamer lens (ICL) implantation has grown in recent years, whether their LD has undergone procedure-induced changes has attracted much attention [6, 7]. Long-term follow-up of LD can reflect the state of aqueous fluid and the risk of anterior capsule opacification after ICL implantation [8, 9]. Consequently, it is of great clinical significance to establish an objective and convenient way to dynamically observe changes in the density of the cornea and lens.

Pentacam, based on a rotating Scheimpflug photography system, obtains multiple images of the ocular anterior segment and computes a three-dimensional corneal map. It can provide an evenly focused representation of the cornea and a section of the lens as compared to slit-lamp photographs [10]. The technique also allows for three imaginary perpendicular planes: the lens, the image, and the subject. Therefore, Pentacam has an extended depth of focus and provides images with sharp resolution [11]. Moreover, Pentacam can provide a series of biological parameters of the anterior segment, including a built-in system that can measure the optical density of the cornea and lens based on the pixel intensity of the obtained images. Its densitometry measurements have been reported to exhibit good accuracy and repeatability [12].

CASIA2 is the latest swept-source anterior segment optical coherence tomography (AS-OCT) instrument. It can facilitate the observation and analysis of the cornea, anterior chamber, and intraocular lens using a wavelength of 1310 nm with a speed of 50,000 axial scans per second [13], boasting a faster scanning velocity, a broader scanning scope, and higher resolution of images. For CASIA2, each 3D image is composed of 512 A-scans and 128 B-scans; a depth of 11 mm and a width of 16 mm are achieved in the scanning scope [14]. CASIA2 has been compared with many other anterior segment imaging devices, such as Spectralis, Anterion, and Visante, to evaluate its repeatability, reproducibility, and inter-device agreement [15–17]. As one of the widely used measurement tools for the anterior segment, Pentacam has often been chosen as the gold standard for agreement evaluation with CASIA2 [18], and studies have been conducted to compare the anterior chamber depth (ACD) [19], corneal thickness, corneal curvature [20], and angle-to-angle distance [14] using these two devices in both healthy participants and patients with certain ophthalmic diseases. Good agreement was found in the measurement of the most anterior-segment biometrics.

However, no studies have compared the accuracy of corneal and lens densitometry between Pentacam and CASIA2. This study aimed to investigate the feasibility of corneal and lens densitometry measurements using CASIA2 and its agreement with Pentacam in myopic participants, in the hope of providing suggestions for ophthalmologists in the assessment and follow-up of patients undergoing refractive surgery and ICL implantation.

## Materials and methods

### Participants

This cross-sectional study included 106 eyes from 53 patients (42 eyes from 21 men and 64 eyes from 32 women, mean age: 26.79 ± 5.08 years), who were recruited at the Eye & ENT Hospital of Fudan University between August and September 2022. The inclusion criteria were as follows: (1) age ≥ 18 years; and (2) no contact lens use for at least 2 weeks or rigid gas permeable contact lens use for at least 4 weeks. The exclusion criteria were as follows: (1) history of progressive corneal dystrophy, cataract, glaucoma, uveitis, retinopathy, or other ocular diseases; (2) history of ocular surgery or trauma; (3) history of systemic diseases or severe psychological diseases; and (4) current use of psychiatric medication, immunosuppressants, glucocorticoids, or any other medication that could affect CD and LD.

#### Examinations

Complete ophthalmologic examinations were performed on all participants to assess the following: (1) uncorrected distance visual acuity (UDVA), refraction sphere (RS), refraction cylinder (RC), axis, spherical equivalent (SE), and corrected distance visual acuity (CDVA) (RT-5100; Nidek Technologies, Japan); (2) non-contact intraocular pressure (IOP) measurement (Canon Full Auto Tonometer TX-F; Canon, Inc., Tokyo, Japan), axial length (AL) measurement (IOLMaster 500, Carl Zeiss Meditec AG, Germany); (3) slit-lamp examination and fundus examination; and (4) corneal thickness (CT), corneal diameter white-to-white (WTW), anterior chamber depth (ACD), and keratometry (Oculus Pentacam HR, Oculus Optikgerate Wetzlar, Germany).

**Fig. 1:**
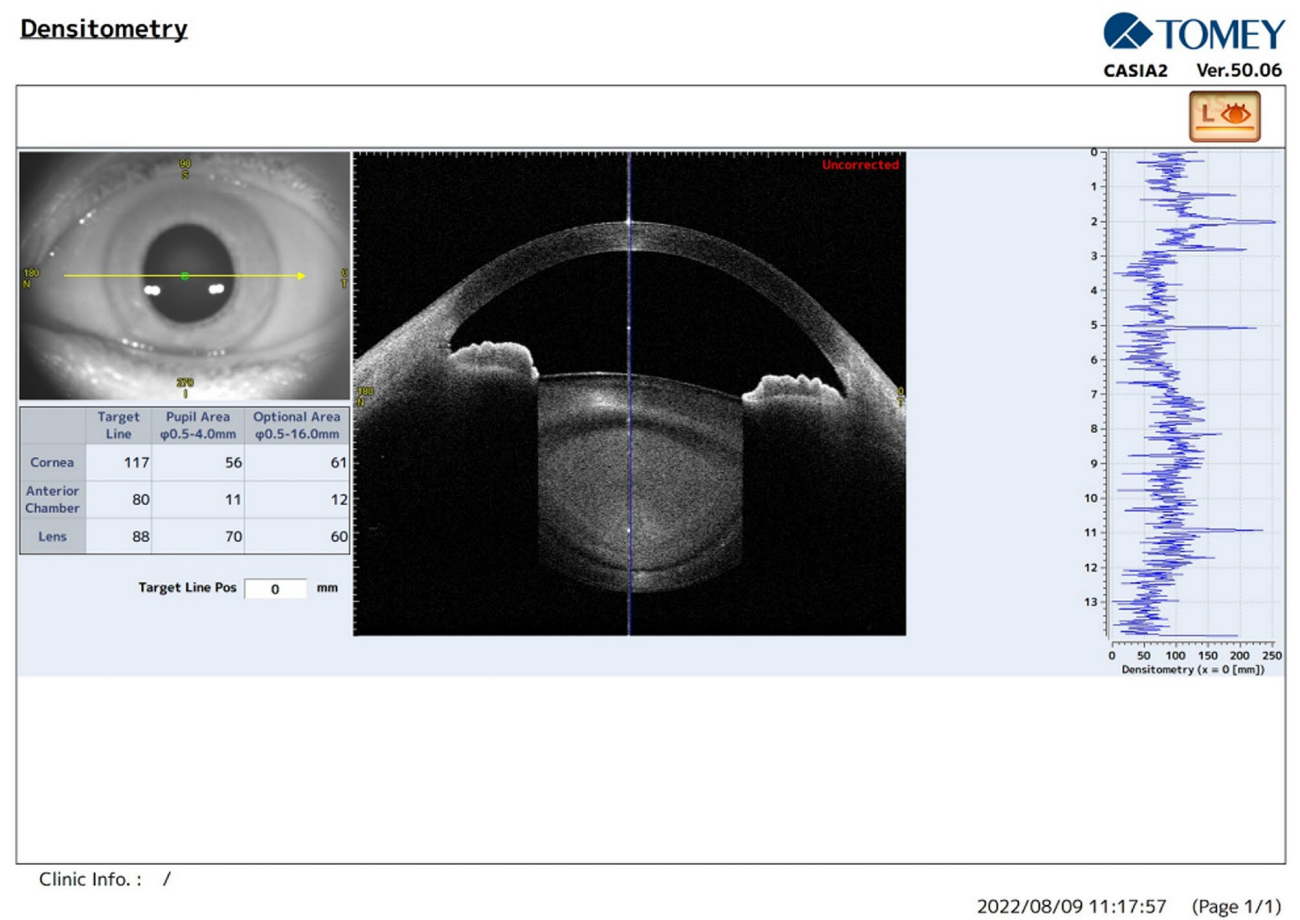
The densitometry analysis of CASIA2

CD and LD were examined using the Pentacam and CASIA2 by two skilled examiners. Both examinations were performed in the same order in a dark room, with CASIA2 following Pentacam after 10 min of rest. Both examinations were performed in the absence of mydriasis. When using Pentacam, the CD values were calculated in four different zones: the central zone was a circle of 2 mm-diameter and centered on the center of the cornea. The second, third, and fourth zones were three concentric annuli extending from diameters of 2–6 mm, 6–10 mm, and 10–12 mm [21]. For illustration, we named these zones as follows: cornea 0–2 mm, cornea 2–6 mm, cornea 6–10 mm, and cornea 10–12 mm. Finally, the average density value of the 12-mm-diameter area was also calculated and named as cornea 0–12 mm. The LD was calculated based on the pixel intensity of the Schiempflug image in a zone with a 3-mm diameter around the pupil center at a 1.5 mm depth [6]. In CASIA2, the CD was measured under the “Anterior Segment”-“Corneal Map” mode, and the LD was measured under the “Pre-Op Cataract”-“Lens Biometry” mode. The built-in software in densitometry analysis allows customized setting of the examined area. Therefore, the measurement of CD and LD by CASIA2 was set and designed in the same way as described above to match Pentacam. The interface of the densitometry analysis of CASIA2 is shown in Fig. 1.

### Statistical analysis

In CASIA2 and Pentacam, the range of densitometry results was 0-250 and 0-100, respectively. In order to compare the results between two instruments, the output of densitometry was transformed to the percentage of grayscale (%grayscale = actual densitometry results / the maximum value of densitometry readings of each instrument * 100%), which define a minimum light scatter of 0% (maximum transparency) and maximum light scatter of 100% (maximum opacity) [21]. To account for the correlation between fellow eyes, generalized linear model (GLM) was used to compare density values between different measurement methods and different subgroups. The repeatability of the two measurements for CD and LD was evaluated using intraclass correlation coefficients (ICCs) (consistency, two-way random effects model). According to McGraw and Wong’s theory [22], an ICC value > 0.7 is considered a sign of very good agreement, while ICC values of 0.4–0.7 and < 0.7 indicate good agreement and poor agreement, respectively. Bland-Altman plots were used to visualize the differences between the CD and LD readings of the two devices. The 95% confidence intervals (CIs) of the limits of agreement (LOAs) were calculated for the upper and lower levels of agreement. Hierarchical linear regression (OD/OS as a hierarchical factor to account for the correlation between fellow eyes) and Pearson correlation analyses were used to quantify the linear correlation between the two sets of values obtained from the two devices. All participants were further classified into subgroups according to age (age < 30 vs. age ≥ 30) and SE (low and moderate myopia: -6.0 D < SE ≤ -0.25 D vs. high myopia: SE ≤-6.0 D). The cut-off value of -6.0 D was based on the definition proposed by American Academy of Ophthalmology [23]. Statistical significance was set at p < 0.05 for all tests. Statistical analyses were performed using SPSS (version 25.0, IBM Corp., Armonk, NY, USA).

## Results

The demographic and corneal characteristics of the participants are shown in Table 1. Examinations were successfully completed in all participants.

**Table 1:** The demographics and corneal characteristics of enrolled patients

Characteristics	Mean ± SD	Range
Age (years)	26.79 ± 5.08	[18, 41]
Gender (male/female)	21/32	
Axial length (mm)	25.93 ± 0.93	[22.15, 27.68]
Refraction sphere (D)	-5.33 ± 1.77	[-8.75, -0.5]
Refraction cylinder (D)	-1.12 ± 0.90	[-3.5, 0]
Spherical equivalent, SE (D)	-5.89 ± 1.80	[-9.5, -1]
CCT (µm)	531.18 ± 33.28	[442, 600]
ACD (mm)	3.17 ± 0.23	[2.45. 3.75]
WTW (mm)	12.11 ± 0.47	[11.10, 14.20]

Abbreviation: SE, spherical equivalent; IOP, intraocular pressure; CCT, central corneal thickness; ACD, anterior chamber depth. WTW, white to white

### Density values of cornea and lens

**Fig. 2:**
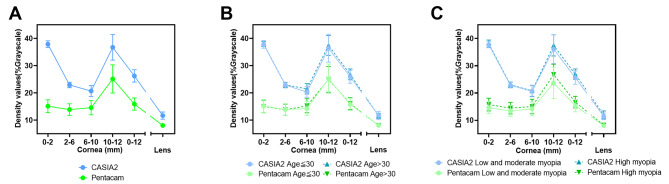
Corneal and lens density values measured by CASIA2 and Pentacam. **(A)** Density values of all participants. **(B) **Density values of patients stratified by age. **(C)** Density values of patients stratified by myopia degree

**Fig. 3:**
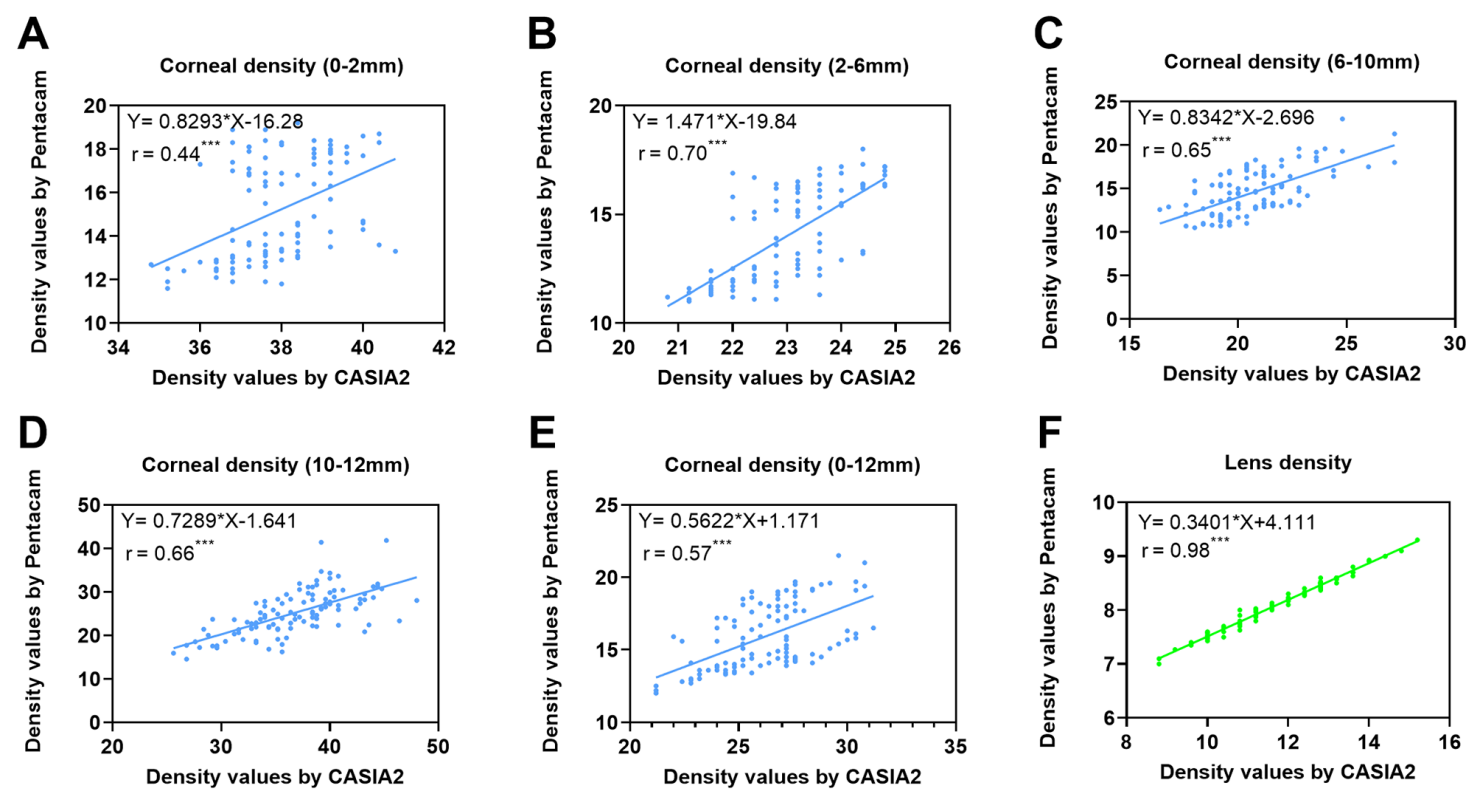
Linear regression analysis for CASIA2 and Pentacam. **(A) **Cornea 0-2 mm, **(B)** Cornea 2-6 mm, **(C)** Cornea 6-10 mm,** (D)** Cornea 10-12 mm, **(E) **Cornea 0-12 mm, **(F)** Lens *: P < 0.05; **: P < 0.01; ***: P < 0.001

The optical density values of different regions of the cornea and lens obtained using the two devices are shown in Table 2; Fig. 2. The results of GLM suggested that after adjusting the influence of fellow eyes, CD and LD obtained from Pentacam and CASIA2 were significantly different. Moreover, CD and LD were higher in HM group than in LMM group (Supplementary Table 1). The difference was smallest in LD, with a median difference of 3.5% in grayscale, followed by cornea 6–10 mm (6.6%), cornea 2–6 mm (9.6%), cornea 10–12 mm (11.75%), and cornea 0–2 mm (23.25%). The median difference in overall CD (0–12 mm) was 10.5%. Among both CASIA2 and Pentacam-measured values, the CD appeared to be higher in the peripheral zone and lower in the paracentral zone (2–6 mm, 6–10 mm). In Pentacam, the density of the central zone (0–2 mm) was similar to that of the paracentral zone. However, the density of the central cornea (0–2 mm) was extraordinarily high when measured by CASIA2, generating a large gap in readings between the two devices. Pearson correlation analyses showed a significant positive correlation between CASIA2 and Pentacam densitometry readings in all regions of the cornea and lens (p < 0.05). A significant strong linear dependence was observed for the LD obtained using CASIA2 and Pentacam (Table 2, r = 0.98). In the measurement of CD, the Pearson correlation was relatively weak (cornea 0–2 mm: r = 0.44; cornea 2–6 mm: r = 0.70; cornea 6–10 mm: r = 0.65; cornea 10–12 mm: r = 0.66; cornea 0–12 mm: r = 0.57). Details of linear regression analyses are shown in Fig. 3.

**Table 2:** Corneal density and lens density measured by CASIA2 and Pentacam

	CASIA2		Pentacam		Pearson correlation	
Optical density (%GSU)	mean ± SD	range [min, max]	mean ± SD	range [min, max]	r	P
Cornea 0-2 mm	37.89 ± 1.25	[34.80, 40.80]	15.15 ± 2.34	[11.60, 19.20]	0.44	< 0.001
Cornea 2-6 mm	22.93 ± 1.00	[20.80, 24.80]	13.90 ± 2.12	[11.00, 18.00]	0.7	< 0.001
Cornea 6-10 mm	20.71 ± 2.04	[16.40, 27.20]	14.59 ± 2.62	[10.50, 23.00]	0.65	< 0.001
Cornea 10-12 mm	36.75 ± 4.71	[25.60, 48.00]	25.15 ± 5.17	[14.50, 41.80]	0.66	< 0.001
Cornea 0-12 mm	26.24 ± 2.29	[21.20, 31.20]	15.92 ± 2.26	[12.00, 21.50]	0.57	< 0.001
Lens	11.69 ± 1.34	[8.80, 15.20]	8.08 ± 0.46	[7.00, 9.30]	0.98	< 0.001

Abbreviation: GSU, grayscale unit

### Agreement test

**Fig. 4:**
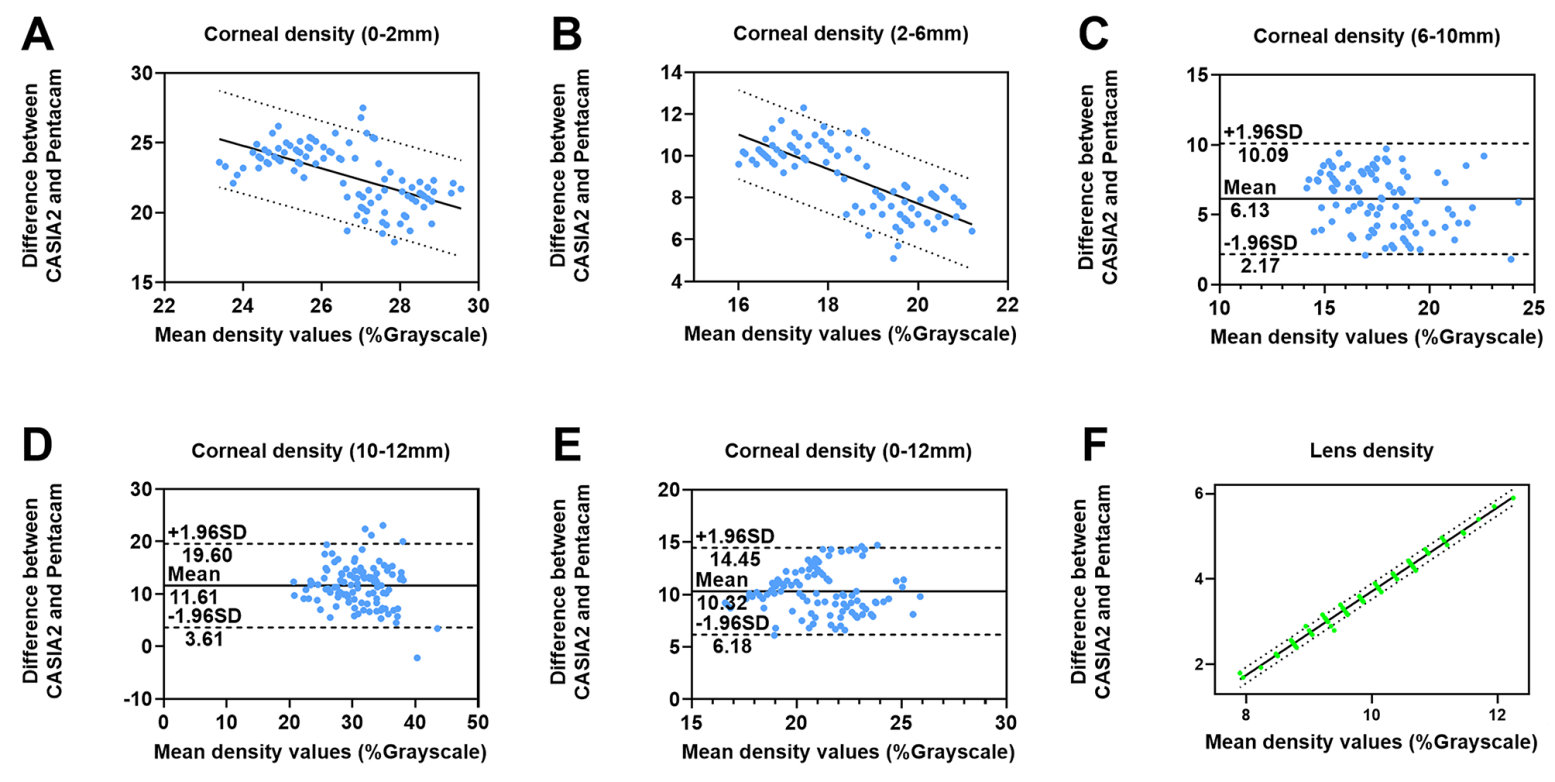
Bland-Altman plots for CASIA2 and Pentacam. The mean values and 95% limits of agreement (LOA) are indicated by solid and dashed lines, respectively. **(A)** Cornea 0-2 mm,** (B)** Cornea 2-6 mm, **(C)** Cornea 6-10 mm, **(D)** Cornea 10-12 mm, **(E)** Cornea 0-12 mm, **(F)** Lens

The agreement of densitometry obtained from CASIA2 and Pentacam is presented in the form of ICC values and Bland-Altman plots in Table 3; Fig. 4. The ICC values were all above 0.7, except for the measurement of the cornea 0–2 mm zone, suggesting good general agreement between the two devices. As the scanning scope shifted peripherally, the ICC gradually increased. The highest ICC occurred in the measurement of the cornea 10–12 mm zone. Overall, the CD and LD measurements showed similar degrees of agreement (CD: 0.726; LD: 0.757). The differences between results from two methods were plotted in Bland-Altman plots (Fig. 4). Due to existence of proportional bias yet homoscedasticity in CD 0-2 mm, CD 2-6 mm and LD, the 95% prediction intervals disposed symmetrically on either side of the straight line of best fit were used to replace the classical LOA. They could indicate the range within which a new observation would be expected to lie, hence prediction intervals were analogous to classical LOA in such cases [24, 25] (Fig. 4A and B F). In cases where there is no apparent proportional bias, i.e., CD at 6–10 mm, 10–12 mm and 0–12 mm, the width of LOA was 7.92%, 15.99% and 8.27%, respectively (Fig. 4, unit: % grayscale). In CD of 0–2 mm and 2–6 mm, the width of prediction intervals was 6.75% and 4.17%, respectively. The percentage of points falling outside the LOA (or prediction limits) in the measurement of different corneal regions and the lens was as follows: 3.77% in cornea 0–2 mm, 5.66% in cornea 2–6 mm, 1.89% in cornea 6–10 mm, 5.67% in cornea 10–12 mm, 2.83% in cornea 0–12 mm, and 3.77% in the lens. The average percentage of all points falling outside the LOA was 3.93%.

**Table 3:** Inter-device agreement as determined by ICC

	CASIA2 and Pentacam		
	ICC value	95% CI	p-value
Cornea 0-2 mm	0.540	[0.324, 0.687]	< 0.001
Cornea 2-6 mm	0.700	[0.559, 0.796]	< 0.001
Cornea 6-10 mm	0.773	[0.667, 0.846]	< 0.001
Cornea 10-12 mm	0.796	[0.700, 0.861]	< 0.001
Cornea 0-12 mm	0.726	[0.597, 0.813]	< 0.001
Lens	0.757	[0.642, 0.834]	< 0.001

### Subgroup analysis stratified by age and SE

**Fig. 5:**
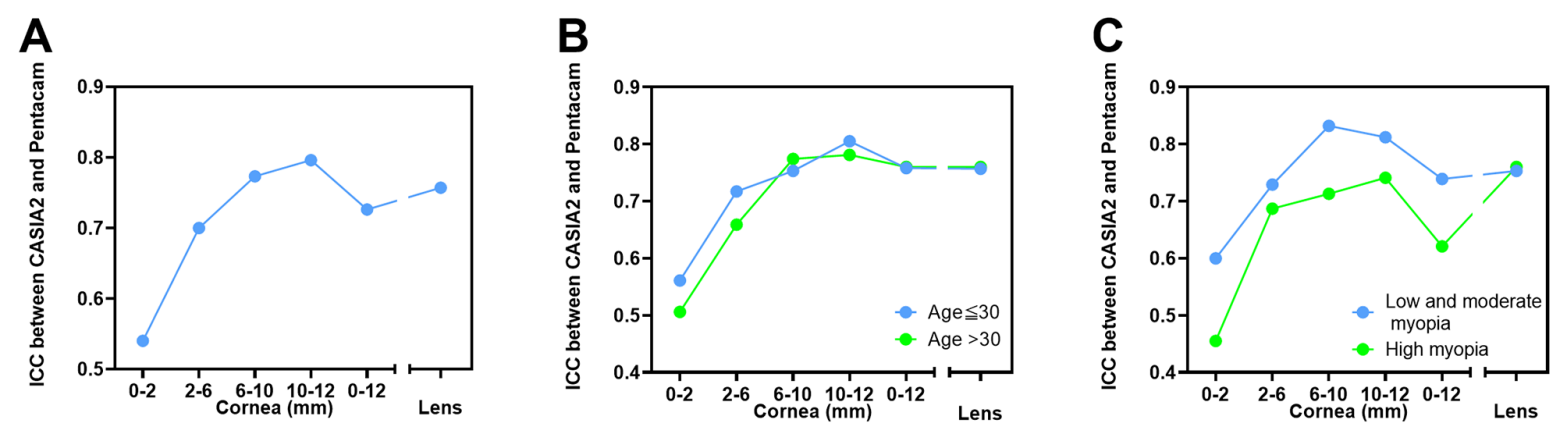
Agreement analysis of CASIA2 and Pentacam in all participants **(A)** and in subgroups stratified by age **(B)** and SE **(C)**

Agreement between the two devices was evaluated in each subgroup; the ICC values are presented in Table 4; Fig. 5. In the ≤ 30 and > 30 years age groups, the ICC values of different measurement scopes were similar, with relatively poor agreement in the central cornea and very good agreement in the peripheral cornea. When comparing the low-to-moderate myopia (SE >-6.0D) with high myopia (SE ≤-6.0D) groups, superiority in agreement was observed in the low-to-moderate myopia group as the ICCs were unexceptionally slightly higher in patients with low-to-moderate myopia (cornea 0–2 mm: 0.600 vs. 0.455; cornea 2–6 mm: 0.729 vs. 0.687; cornea 6–10 mm: 0.832 vs. 0.713; cornea 10–12 mm: 0.812 vs. 0.741; cornea 0–12 mm: 0.739 vs. 0.621). However, regardless of age and SE, the ICC of LD measurement between the two devices remained stable at the level of 0.75–0.76 in all subgroups.

**Table 4:** Inter-device agreement as determined by ICC in subgroups stratified by age and SE

ICC	Age ≤ 30	Age > 30	Low and moderate myopia (-6.0D < SE≤-0.25 D)	High myopia (SE≤-6.0D)
Cornea 0-2 mm	0.561	0.506	0.600	0.455
Cornea 2-6 mm	0.717	0.659	0.729	0.687
Cornea 6-10 mm	0.753	0.774	0.832	0.713
Cornea 10-12 mm	0.805	0.781	0.812	0.741
Cornea 0-12 mm	0.758	0.760	0.739	0.621
Lens	0.757	0.760	0.753	0.760

Note: P-values of all subgroup ICC analysis are smaller than 0.001, except for Cornea 0-2 mm in Age > 30 group (p = 0.03), Cornea 0-2 mm in high myopia group (p = 0.018)

## Discussion

Densitometry of the cornea and lens is of great clinical significance. It can reflect the overall condition and local lesions of the examined areas. The measurement of CD and LD has undergone several upgrades, from slit-lamp examination and ultrasound biomicroscopy to Schiempflug imaging.

This is the first study to compare the measurement of CDs and LDs between CASIA2 and Pentacam. Prior studies have reported the results of corneal densitometry using Scheimpflug optical assessment. Otri et al. reported that when measured by Pentacam, the normal density value of the total cornea was 12.99 ± 2.58%grayscale, with the range of 4.7–22.0 in healthy corneas [1]. Dhubhghaill reported that densitometry values of the cornea were lowest in the central zone (16.76 ± 1.87%grayscale) and highest in the periphery (27.36 ± 7.47%grayscale) in healthy participants, while the surrounding 2–6 mm annulus had very similar densitometry values to the central zone [21]; notably, these results are in accordance with our findings from Pentacam data. The Pentacam-Scheimpflug image system has been used to investigate lens densitometry in many published studies [26] [27]. Weiner et al. reported that lens densitometry based on Scheimpflug imaging was highly repeatable in eyes without cataracts [26]. In a prospective study on LD conducted by Bayrak et al., the average LD of healthy participants measured by Pentacam in the control group was 8.3 ± 0.9% grayscale [27]. The range of LDs in our study approximated to what were reported in previous studies.

In this study, we found good agreement in the measurements of the average density of the cornea and lens using CASIA2 and Pentacam. However, the density values of the two devices were not directly interchangeable and comparable, even after the percentage conversion. The two sets of data generated by CASIA2 and Pentacam were of a linear correlation relationship with random bias. Thus, consistency ICC instead of absolute agreement ICC was used in the study. Except for cornea 0–2 mm, the ICC values of all other zones of the cornea and lens were above 0.7, suggesting a good level of consistency. The weaker agreement of the central corneal density was caused by the paradoxically high CD in the 0–2 mm cornea obtained from CASIA2. CD has been shown to be higher in the periphery compared to the central by Pentacam measurement [21, 28]. We believed the abnormally high central CD was an artifact related to the central position of CASIA2’s light source and its reflection. In the cross-sectional image generated by CASIA2, a bright line with strong reflection along the optical path was clearly visible, passing through the central corneal (Fig. 1). This would greatly affect the results of CD 0–2 mm, which was calculated based on the grayscale of image. Therefore, CASIA2 measurement of central corneal density was rather overestimated and deviated from its actual value, hence it must be considered with caution.

In all zones of the cornea and lens, the density values obtained by CASIA2 were higher than those obtained by Pentacam, which could be caused by the different approaches of the two devices for anterior segment imaging. CASIA2 is a swept-source OCT (SS-OCT), a high-resolution tomographic and biomicroscopic device used for in vivo imaging and measurement of ocular structures in the anterior segment. SS-OCT relies on backscattered light compared to a reference beam and employs Fourier transformation for image reconstruction [29]. Pentacam is a Scheimpflug imaging-based device that uses a rotating camera to capture multiple images of the anterior segment and generate 3D images [30]. Although the densitometry analyses of the two devices are both based on the grayscale of the captured images, their principles of imaging differ greatly. As a result, there were noticeable differences in terms of image quality and density values of the cornea and lens obtained from CASIA2 and Pentacam. The densitometry analysis software for these two devices also provides different user experiences. CASIA2’s densitometry boasts customization of the scanning scope width, automated density calculation and analyses, and simultaneous measurement of the cornea, lens, and anterior chamber under one scanning mode. In comparison, Pentacam’s densitometry slightly fails in flexibility, simplicity, and precision because of the unchangeable preset parameters of CD measurement and manual operation of LD measurement, which requires users to choose one or more Schiempflug images with relatively good quality and to select the zones of the lens either by entering parameters or shifting the marquee.

In Bland-Altman analyses, LOA was constructed to help determine whether the agreement between two methods was sufficiently close for them to be interchangeable. Based on previous reports of corneal density in pathological conditions, total cross-sectional CD increased to 58.4 ± 19.5%grayscale during acute infection and returned to 33.4 ± 17.3%grayscale after complete resolution, whilst the healthy cornea had a density of 12.99 ± 2.58%grayscale [1]. There was a large gap in CD values between healthy and infected corneas. In other conditions, such as keratoconus accompanied with Down syndrome, the difference in the CD between patients and healthy controls was smaller (19.35 ± 2.92% vs. 15.78 ± 2.67%) [31]. We believed this was related to the nature of each keratopathy. In diseases where change in density was one of the defining pathologic manifestations, the possibility of misdiagnosing keratopathy due to inter-equipment errors in CD was relatively low. In other diseases where the change in CD was more subtle, typically more comprehensive examinations were required to arrive at a diagnosis. Therefore, we believed the LOA of CD was considered clinically acceptable in the study, if interpreted with other examination results and clinical evidence. In terms of LD, the prediction interval was quite narrow, and the strong linear correlation between two sets of data facilitated the inter-equipment transformation of densitometry readings.

In addition, although Pentacam is one of the most widely used devices in the assessment of ocular anterior segment, it is restricted by pupil diameter and its limited depth of scanning scope when measuring the LD. While CASIA2 has a scanning scope of 11 mm in depth and 16 mm in width [14], its lens densitometry analysis takes into account the periphery of the lens. Since the opacity of the lens usually starts in the peripheral lens after ICL implantation [32], peripheral LD is considered of great clinical value. In addition, CASIA2 could reflect more minor and earlier changes in CD and LD; hence, its superior sensitivity could remind ophthalmologists of trivial pathological signs that may otherwise be overlooked. Although the absolute numeric density values of the two devices were not interchangeable owing to differences in imaging principles, the two sets of data presented the same tendency and a certain degree of linear association. Therefore, we believed that CASIA2’s densitometry could indicate the relative level of CD and LD and distinguish obvious abnormal density values in pathological conditions. Nevertheless, the literature on CD and LD measured by CASIA2 is rather scant. We suggested that CASIA2’s densitometry results be mathematically transformed before they were subjected to interpretation based on knowledge or understandings from Pentacam’s densitometry results, until further studies establish more accurate diagnostic standards that are tailored for CASIA2’s densitometry.

In this study, the inter-device agreement between CASIA2 and Pentacam densitometry did not appear to be affected by age. Intriguingly, we found that the agreement of CD measurements was poorer in the high myopia subgroup. This can be partly explained by poorer fixed vision during examination in the high myopia group, which could result in a larger scope of the central cornea affected by the light beam of CASIA2, resulting in a greater systematic bias. On the other hand, since the CD values were higher in the high myopia group than in the mild-to-moderate myopia group in our findings, the increase in measurement values could cause the two sets of data to diverge due to a scaling effect, resulting in a lower ICC and weaker agreement. Similar scaling effects have been noted in previous studies comparing AS-OCT measurements between the Spectralis, CASIA2, and Cirrus [15, 33]. One hypothesis is that the systematic effect originates from how OCT devices account for corneal refraction, which is a parameter used to scale the corresponding OCT B-scans [15].

This study had a few limitations. First, all densitometric measurements were obtained without mydriasis. This limited the scanning scope of the lens, and only the central and anterior regions of the lens were included in the density calculations. Second, analysis of CD at different depths and layers was not included in this study. The average density values of the entire corneal layer were adopted and compared between the two devices. Third, the study included both eyes of participants in the analyses to maximize the sample size and retain more information. However, the correlation between fellow eyes could potentially result in underestimated p values and narrower confidence intervals [34]. Despite the use of generalized linear model and hierarchical regression method to account for such correlation, the influence of including both eyes in the study should be noted by readers. Finally, the study enrolled healthy participants who had no ocular conditions other than myopia. The measurement of density using CASIA2 in patients with other ocular diseases, such as cataract, requires further evaluation.

## Conclusion

Our results demonstrated that the readings of CASIA2 were generally higher than those of Pentacam, making the results from two devices uninterchangeable; however, the two sets of data exhibited the same tendency and linear correlation. In general, CASIA2 provides a reliable way of measuring the density of the cornea and lens with clinically acceptable agreement with Pentacam, except that the readings of CD in the central corneal zone and in highly myopic patients must be interpreted with caution.

### Electronic supplementary material

Below is the link to the electronic supplementary material.

Supplementary Material 1

## Data Availability

The datasets generated and/or analyzed during the current study are not publicly available due to funding requirement but are available from the corresponding author on reasonable request.
