# Supplementary material for: Comparison of corneal and lens density measurements obtained by Pentacam and CASIA2 in myopes
Source: BMC Ophthalmol. 2023 Nov 10;23:448. doi: 10.1186/s12886-023-03199-3 (PMC10636911; doi:10.1186/s12886-023-03199-3)
Supplement: Supplementary file 1 — Supplementary Material 1 [file 12886_2023_3199_MOESM1_ESM.docx]

Supplementary Table 1. Generalized linear models of corneal and lens density.

|  |  | Cornea  0-2mm | Cornea  2-6mm | Cornea  6-10mm | Cornea  10-12mm | Cornea  0-12mm | Lens |
| --- | --- | --- | --- | --- | --- | --- | --- |
| Instruments | B | -22.746 | -9.028 | -6.13 | -11.606 | -10.315 | -3.602 |
| CASIA2 vs Pentacam | p | **<0.001** | **<0.001** | **<0.001** | **<0.001** | **<0.001** | **<0.001** |
| Age group | B | -0.16 | -0.121 | -1.243 | -0.911 | -0.871 | -0.009 |
| Age≤30 vs Age>30 | p | 0.561 | 0.62 | **<0.001** | 0.203 | **0.006** | 0.951 |
| SE group | B | -0.767 | -0.6 | -0.719 | -2.325 | -1.437 | -0.373 |
| LMM vs HM | p | **0.003** | **0.009** | **0.023** | **0.001** | **<0.001** | **0.007** |
| Eyes | B | -0.192 | -0.149 | -0.312 | -0.792 | -0.39 | -0.169 |
| OD vs OR | p | 0.446 | 0.504 | 0.314 | 0.227 | 0.183 | 0.208 |

P value <0.05 is highlighted in bold.
